# Supplementary material for: The causal role of circulating inflammatory markers in osteoporosis: a bidirectional Mendelian randomized study
Source: Front Immunol. 2024 Jul 18;15:1412298. doi: 10.3389/fimmu.2024.1412298 (PMC11291241; doi:10.3389/fimmu.2024.1412298)
Supplement: Supplementary file 1 [file DataSheet_1.zip › supplementary material/CIM-OSTEOPOROSIS /GCST90274790.csv_leave_one_out.pdf]

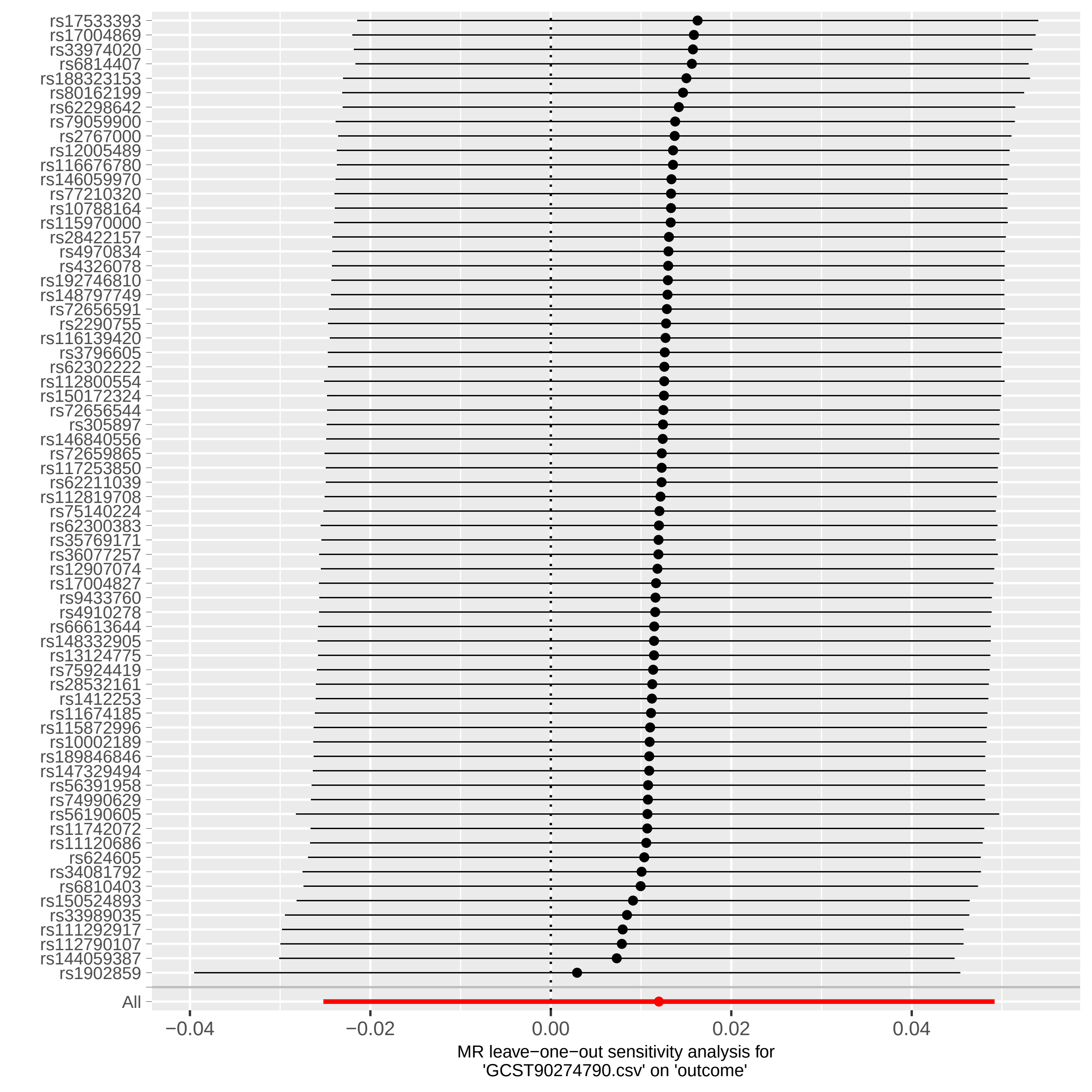

rs17533393
rs17004869
rs33974020
rs6814407
rs188323153
rs80162199
rs62298642
rs79059900
rs2767000
rs12005489
rs116676780
rs146059970
rs77210320
rs10788164
rs115970000
rs28422157
rs4970834
rs4326078
rs192746810
rs148797749
rs72656591
rs2290755
rs116139420
rs3796605
rs62302222
rs112800554
rs150172324
rs72656544
rs305897
rs146840556
rs72659865
rs117253850
rs62211039
rs112819708
rs75140224
rs62300383
rs35769171
rs36077257
rs12907074
rs17004827
rs9433760
rs4910278
rs66613644
rs148332905
rs13124775
rs75924419
rs28532161
rs1412253
rs11674185
rs115872996
rs10002189
rs189846846
rs147329494
rs56391958
rs74990629
rs56190605
rs11742072
rs11120686
rs624605
rs34081792
rs6810403
rs150524893
rs33989035
rs111292917
rs112790107
rs144059387
rs1902859
All
−0.04
−0.02
0.00
0.02
0.04
MR leave−one−out sensitivity analysis for
'GCST90274790.csv' on 'outcome'
